# Branched Polyurethanes Based on Synthetic Polyhydroxybutyrate with Tunable Structure and Properties

**DOI:** 10.3390/polym10080826

**Published:** 2018-07-26

**Authors:** Joanna Brzeska, Anna Maria Elert, Magda Morawska, Wanda Sikorska, Marek Kowalczuk, Maria Rutkowska

**Affiliations:** 1 Department of Commodity Industrial Science and Chemistry, Gdynia Maritime University, 83Morska Street, 81-225 Gdynia, Poland; m.morawska@wpit.am.gdynia.pl (M.M.); m.rutkowska@wpit.am.gdynia.pl (M.R.); 2 Nanotribology and Nanostructuring of Surfaces, Federal Institute for Materials Research and Testing (BAM), Unter den Eichen 87, 12205 Berlin, Germany; anna-maria.elert@bam.de; 3 Centre of Polymer and Carbon Materials, Polish Academy of Sciences, 34 M. Curie-Sklodowska Street, 41-819 Zabrze, Poland; wsikorska@cmpw-pan.edu.pl; 4 School of Biology, Chemistry and Forensic Science, Faculty of Science and Engineering, University of Wolverhampton, Wolverhampton WV1 1SB, UK

**Keywords:** polyurethane structure, linear and branched polyurethanes, synthetic polyhydroxybutyrate, thermal properties, sorptive properties

## Abstract

Branched, aliphatic polyurethanes (PURs) were synthesized and compared to linear analogues. The influence of polycaprolactonetriol and synthetic poly([R,S]-3-hydroxybutyrate) (R,S-PHB) in soft segments on structure, thermal and sorptive properties of PURs was determined. Using FTIR and Raman spectroscopies it was found that increasing the R,S-PHB amount in the structure of branched PURs reduced a tendency of urethane groups to hydrogen bonding. Melting enthalpies (on DSC thermograms) of both soft and hard segments of linear PURs were higher than branched PURs, suggesting that linear PURs were more crystalline. Oil sorption by samples of linear and branched PURs, containing only polycaprolactone chains in soft segments, was higher than in the case of samples with R,S-PHB in their structure. Branched PUR without R,S-PHB absorbed the highest amount of oil. Introducing R,S-PHB into the PUR structure increased water sorption. Thus, by operating the number of branching and the amount of poly([R,S]-3-hydroxybutyrate) in soft segments thermal and sorptive properties of aliphatic PURs could be controlled.

## 1. Introduction

Typical linear polyurethane (PUR) is built with (i) a soft segment (based on oligomeric polyester- or polyetherdiol), which gives flexibility and softness of polymer and (ii) a hard segment (synthetized with diisocyanate and low molecular chain extender) the aim of which is to increase stiffness, hardness changes, etc. Immiscibility of soft and hard segments facilitates their reorganization into domains. Further ordering of chains in domains can lead to formation of crystallites. The polar nature of the urethane group influences their tendency to hydrogen bonding. Creation of hydrogen bonds between N–H group and C=O in urethanes facilitates their ordering and phase separation. Whereas hydrogen bonds of the urethane group with oxygen of the soft segment can reduce chains mobility and their ordering [1]. Generally, hydrogen bonds create physical reinforcement of the PUR network which causes increased strength and stiffness [2].

Thermodynamic incompatibility of hard and soft segments and connected with this, their insolubility, cause microphase separation in segmented PURs [3]. Ordering of chains in both separated domains of segments can lead to formation of crystallites.

Branched polymers are a kind of macromolecules, whose architecture is neither linear nor cross-linked. In addition to polymers with long or short side-chains, this group of polymers also includes densely branched structures with a large number of functional end groups (star like and hyperbranched polymers, and dendrimers).

The architecture of branched polymers causes an intermediate structure between cross-linked and linear polymers. The presence of side chains in their structure significantly influences the properties of polymers.

Branched PURs are generally characterized by good solubility in many organic solvents and compatibility with different materials, low viscosity (both in the molten state and in the solution) and they have free spaces inside the network. In consequence, they can be used as drug carriers, catalysts, chemical sensors, coatings, binders, elastomers, sorption mats, etc. [2,4].

In branched polymers linear side-chains diverge (uniformly or randomly) from branching points of the linear flexible chain (backbone). In this case, introducing trifunctional compound into the PUR structure reduces a possibility of chains to order and form soft and hard domains. However, local aggregation of soft or hard segments and their microphase separation can often still occur even in cross-linked PURs [1]. The presence of branching points generally introduces irregularities in the polymer structure and consequently leads to lowered crystallinity and creation of smaller crystals with the lower melting point in comparison to linear polymers [5]. Tendency of urethane groups to create hydrogen bonds is strongly affected by side-chains. Both short and long side-chains can influence a possibility of interaction between polymer chains.

Polyhydroxybutyrate (PHB) is a polymer that naturally exists in prokaryote and eukaryote cells. Biosynthesized PHB is high crystalline, stiff and brittle material, which makes it difficult to use. Therefore, it is valuable to us its synthetic analogue, which can be obtained as an amorphous, elastic polymer.

**Figure 1:**
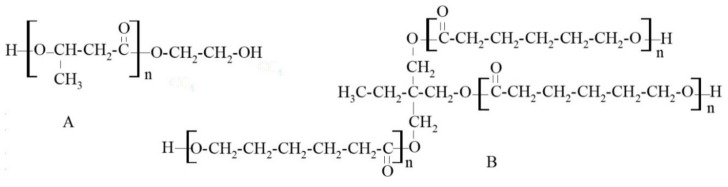
Schematic structure of polyhydroxybutyratediol (**A**) and polycaprolactonetriol (**B**).

Synthetic PHB can be obtained via ring-opening polymerization (ROP) of β-butyrolactone to isotactic, atactic and syndiotactic poly(3-hydroxybutyrate) [6]. Atactic (R,S)-PHB is an amorphous polymer with low glass transition temperature and it maintains elastomer properties at room temperature. Because of the secondary hydroxyl group in the PHB_diol_ structure methyl side-groups are present in its chains (Figure 1A).

Through the right combination of polycaprolactonetriol (PCL_triol_) (Figure 1B) with polycaprolactonediol (PCL_diol_), branched PURs with the highest possible molecular weight without reaching gelation and formed into foils could be obtained [5]. Knowing that introduction of amorphous R,S-PHB influences thermal, sorptive and surface [7] properties of linear and cross-linking PUR materials and their degradability profile [8], such effects are also expected in branched PURs.

In this paper branched PURs, as potential sorptive materials and their linear analogues were comparatively studied. The influence of PCL_triol_ and R,S-PHB presence in the soft segments structure on thermal and sorptive properties of obtained PURs was estimated.

## 2. Experimental Section

### 2.1. Materials

β-butyrolactone (Aldrich, Steinheim, Germany) was purified [9], 18-crown-6 complex (Fluka, Bucharest, Romania), 3-hydroxybutyric acid sodium salt and 2-bromoethanol (Aldrich) were used as received. Oligomerols of R,S-PHB (*M*_n_ 1700), PCL_triol_ (*M*_n_ 900, Aldrich, St. Louis, MO, USA) and PCL_diol_ (*M*_n_ 1870, Aldrich, St. Louis, MO, USA) were dried by heating at 60–65 °C under reduced pressure (1.4 hPa). 4,4’-methylene dicyclohexyl diisocyanate (H_12_MDI) (Aldrich, St. Louis, MO, USA) was vacuum distilled; 1,4-butanediol (1,4-BD) (Aldrich, Steinheim, Germany) was distilled azeotropically with benzene; *N*,*N*-dimethylformamide (DMF) (Chempur, Piekary Śląskie, Poland) was dehydrated over diphosphorous pentoxide (P_2_O_5_) and distilled under low pressure. Catalyst tin(II) octanoate (OSn) (Alfa Aesar, Karlsruhe, Germany) was used as received.

### 2.2. Synthesis of Linear PURs

Oligomerole of telechelic (with OH groups on both sides of chains) poly([R,S]-3-hydroxybutyrate) (R,S-PHB) was obtained by anionic ring opening polymerization of ß-butyrolactone initiated by 3-hydroxybutyric acid sodium salt/18-crown-6 complex at room temperature and terminated with 2-bromoethanol [10]. Obtained R,S-PHB was generally atactic and almost completely amorphous polymer (with very small melting enthalpy about 4 J/g).

The synthesis of PURs was carried out in a two-step reaction with the molar ratio of NCO:OH = 2:1 in the prepolymer step [7]. Prepolymer of PURs was synthesized for 3 h at 70–75 °C under vacuum with oligomer (PCL_diol_ or blend of PCL_diol_ with R,S-PHB) and H_12_MDI in the presence of OSn. NCO-terminated prepolymer was dissolved in DMF, and next its molecular weight was increased by a reaction with the chain extender (1,4-BD) for 2 h at 60 °C. The final molar ratio of NCO:OH in PURs was 1:1. PUR foil was formed by pouring the polymer solution on Teflon plates and heating it at 80–105 °C in the vacuum heater for 6 h.

### 2.3. Synthesis of Branched PURs

Branched PURs were synthesized in the same way as linear PURs. Soft segments were built with PCL_triol_, but PCL_diol_ and R,S-PHB were additionally added to vary a distance between branch points. This branched PUR turned out to be easily soluble in organic solvents (dimethylformamide, toluene, acetone, ethanol and chloroform).

Amounts of reactants in the synthesis of PUR are shown in Table 1.

**Figure 2:**
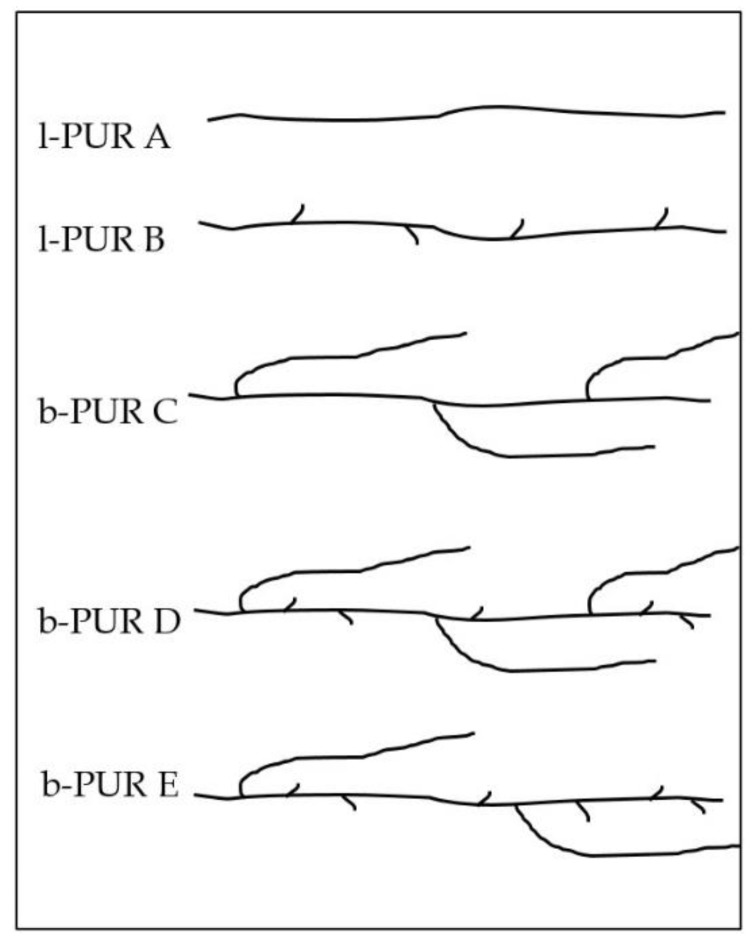
Illustration of the schematic structure of linear and branched PURs.

Substrates used for PURs determined how they were built. All obtained PURs were aliphatic polyesterurethanes, differing in architecture of the structure (Figure 2). Linear PURs differed in side-chain methyl groups from R,S-PHB. Whereas, using PCL_triol_ to build soft segment, introduced branch nodes into the structure of branched PURs. So, they differed in the amount of short (methyl groups from R,S-PHB) and long (polycaprolactone chains from PCL_triol_) side-chains. These lateral branches should affect interactions between chains (e.g., hydrogen bonds creation), which consequently should influence sorption and thermal properties.

### 2.4. Methods

#### 2.4.1. Differential Scanning Calorimetry (DSC)

Thermal properties of PURs and their blends were determined using the Setaram thermal analyzer (Setaram, Caluire, France). Indium and lead were used for calibration. Specimens (with mass about 20 mg) were sealed in aluminum pans and scanned from 20 to 200 °C with the heating rate of 10 °C/min. All experiments were conducted in a flow of dry N_2_.

#### 2.4.2. Raman Spectroscopy

All Raman spectra were recorded in backscattering geometry by a WiTec 300R Alpha device (WiTec, Ulm, Germany). The spectra are shown without background correction. A ruled 600 grooves/mm grating was chosen in the optical spectrometer (WiTEC, UHTS 300), which was equipped with a Peltier-cooled CCD camera (ANDOR, iDus DV401A) operating at a temperature of 210 K. The resulting wave number resolution was <2 cm^−1^. Investigated solids were excited at a power level of 2.5 mW using the 532 nm emission line of a continuous wave laser (Spectra Physics, Excelsior). The Raman-laser radiation was focused on the sample surface with a microscope (Zeiss EC Epiplan 20’, NA 0.4) probing a circular spot of ~4 µm in the diameter. At the given experimental conditions, the Raman-laser induced oxidation processes could be excluded. The spectra were recorded with the integration time of 10 s and 10 accumulations each. After measurement, spectra were background subtracted using WITec Project 2.08 software.

#### 2.4.3. ATR IR Spectroscopy

FTIR investigations of solids were performed using attenuated total reflection (ATR, Smart Orbit Accessory) in a Nicolet 6700 FTIR spectrometer (Bruker Optics, Ettlingen, Germany) with a DTGS KBr detector. To obtain a spectrum, 32 scans were taken at optical resolution of 4 cm^−^^1^. For ATR-FTIR investigations the materials were pressed on the diamond cell to achieve surface sensitive test results.

#### 2.4.4. Density

Density of polymer samples was determined according to ISO 1183 standard and using analytical balance equipped with a density determination kit (Radwag, Radom, Poland).

#### 2.4.5. Oil and Water Sorption

PURs and blends samples were immersed in sunflower oil at 37 °C for 24 h and next they were weighed after wiping off the oil with filter paper [11]. For water sorption estimation, investigated polymers were immersed in deionized water for 14 days at 37 °C. Next the swollen samples were gently blotted with filter paper and weighed. Sorption was calculated (using the gravimetric method) from the weight after incubation (*w_i_*) and the initial weight (*w*_0_) by:

Sorption% = (*w_i_* − *w*_0_)/*w*_0_ × 100%. The results were the average of three measurements.

## 3. Results and Discussion

**Figure 3:**
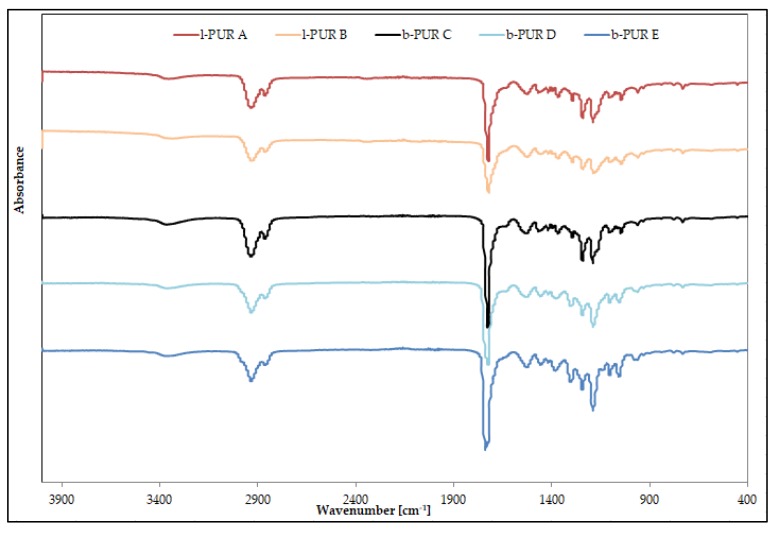
FTIR spectra of linear and branched PURs.

The chemical structure of synthetized PURs was confirmed with FTIR and Raman spectroscopy. Figure 3 shows FTIR spectra of linear and branched PURs.

Infrared spectra of linear and branched PURs were very similar and showed characteristic bands typical for PURs. The absence of bands ascribing functional groups of starting substrates on FTIR spectra indicated completion of the polyaddition reaction.

The region between 3410 and 3200 cm^−1^ was ascribed to N–H stretching vibration, whereas the region between 2990 and 2840 cm^−1^ to C–H stretching vibration (of both soft and hard segments). Infrared absorptions between 1780 and 1620 cm^−1^ indicated the presence of the carbonyl group (amide I), whereas bands 1550–1510 cm^−1^ ascribed deformation vibrations of N–H groups (amide II) and 1242–1240 cm^−1^ C–O stretching of the carbonate group. Bands corresponding to asymmetric and symmetric C–H stretching vibrations of aliphatic –CH_2_ groups were observed at 2905 and 2860 cm^–1^ respectively (Figure 3).

**Figure 4:**
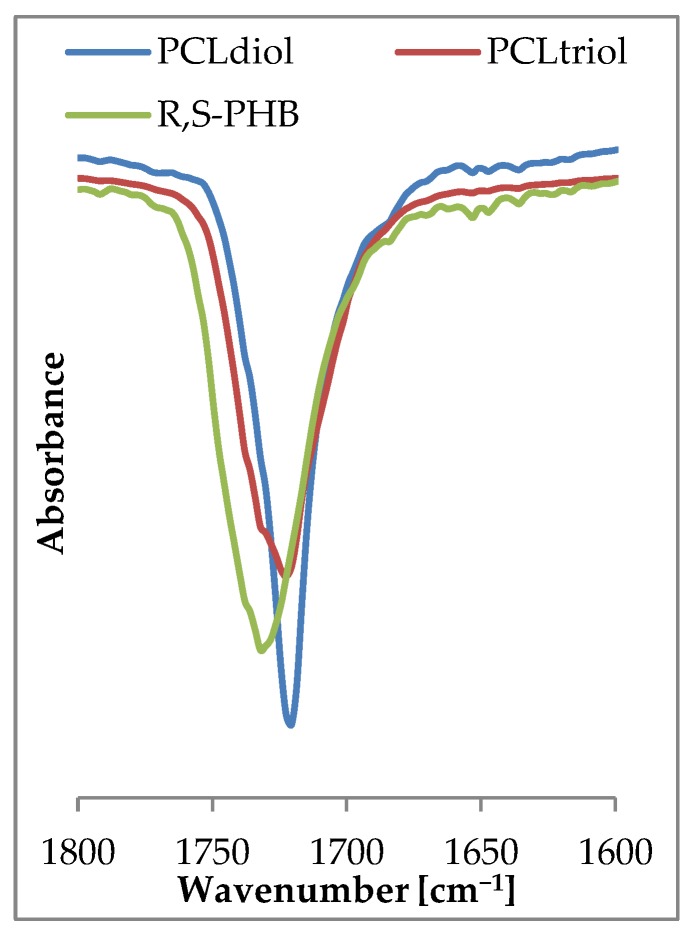
FTIR spectrum of C=O stretching region of oligomeroles used for the PURs synthesis.

Spectra of carbonyl moiety in PURs can be highly complex due to numerous origins of carbonyl and hydrogen bond interactions within the polymer network. The observed on FTIR spectrum of PCL_diol_ (Figure 4) carbonyl peak at 1720 cm^−1^ was narrow and slender, which suggested homogeneous nature of this moiety. The presence of branch nodes in PCL_triol_ and the lateral methyl group in chains of R,S-PHB (only with CH_2_ and CH groups between ester moiety in comparison to five CH_2_ in PCL) changed molecular interaction, causing in these cases the carbonyl peak to be wider and less uniform than for PCL_diol_. Moreover, the peak appeared at a higher wavenumber (1730 cm^−1^).

**Figure 5:**
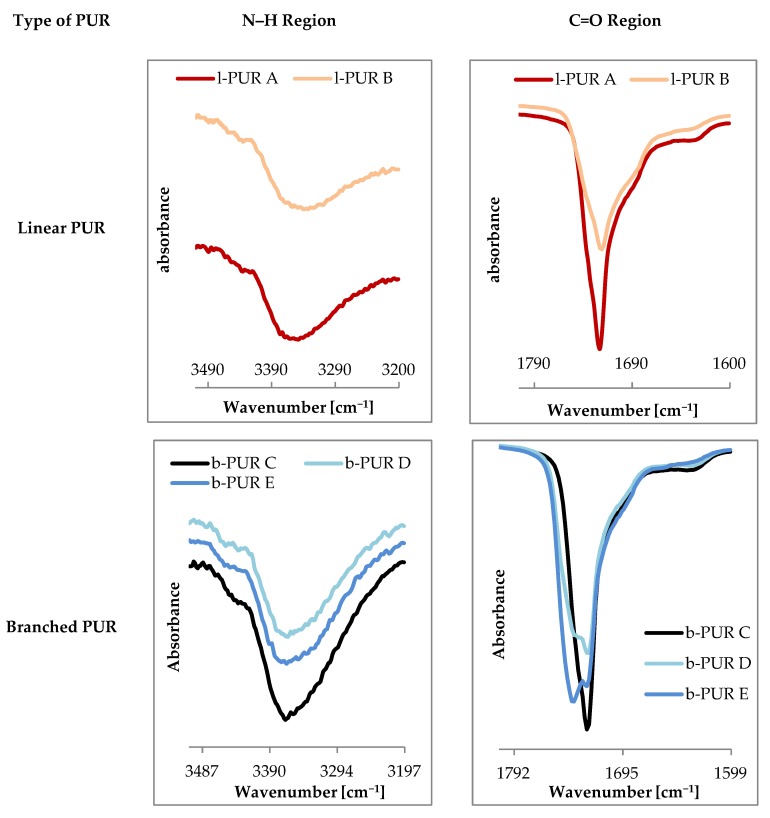
FTIR spectrum of N–H and C=O stretching regions of linear and branched PURs.

On FTIR spectra observed carbonyl bands were a result of overlapping of the peaks of carbonyl moieties from oligomeroles and urethane groups. In the case of linear l-PUR A with soft segments built with only PCL_diol_ frequency (1720 cm^−1^) and shape of the carbonyl stretching peak were identical as in pure PCL_diol_ (Figure 5 compared to Figure 4). Only a small shoulder at right sight of the peak was observed. It was the overlapping band of carbonyl groups from urethane (with wavenumber 1690 cm^−1^). After introducing the R,S-PHB into soft segments the band in the stretching carbonyl region became wider and less sharp. The overlapped urethane C=O peak was also observed on l-PUR B FTIR spectra as a shoulder at lower frequencies (at 1690 cm^−1^) in relation to that of the ester group. This band was almost no discernible at 1720 cm^−1^.

Using PCL_triol_ to build soft segments caused that shape of the carbonyl band to be more complicated (Figure 5). Besides two mentioned peaks at 1740 cm^−1^ another one appeared, which could be attributed to free C=O groups. On FTIR spectra of linear PURs no bands were detected above 3500 cm^−1^ (Figure 5), which suggested that all N–H groups were engaged in formation of hydrogen bonds. However, at 3430–3460 cm^−1^ on FTIR spectra of branched PURs (Figure 5), a little intensity shoulder was observed. It was often described as the overtone of the band at 1720 cm^−1^ [12]. Taking into consideration that small intensity bands indicating the presence of non-bonded C=O stretching of the urethane and carbonate groups, were visible at 1740 cm^−1^ (Figure 5), it was concluded that this peak (at 3430–3460 cm^−1^) ascribed non-associated N-H groups. Moreover, increasing R,S-PHB in the structure of branched PURs inhibited a tendency of urethane groups to hydrogen bonding. In b-PUR E intensity of bands ascribed free C=O ester groups were larger than those bound by hydrogen bonding. Probably, the presence of the side-chain methyl group in the R,S-PHB chain hindered formation of hydrogen bonds. As it was said, the band attributed to stretching vibrations of C=O in the urethane group strongly overlapped with these of the ester group, so no quantitative information about relations between free and bonded ester and urethane groups could be obtained.

The peak observed at 1381 cm^−1^ on FTIR spectra of R,S-PHB (Figure S1) was ascribed as banding deformation vibration of CH_3_ according to Eldessouki et al. [13]. Its presence was noted on FTIR spectra of branched PURs based on R,S-PHB at 1380 cm^−1^. Intensity of the peak was the highest in the case of b-PUR E, because of the high amount of R,S-PHB (Figure 3). On spectra of l-PUR B (obtained with the smaller amount R,S-PHB in comparison to branched PURs) this peak overlapped with C–H bending vibration (Figure 3).

Raman spectroscopy, which is a complementary technique to infrared spectroscopy, also measures vibrational energy levels [14].

**Figure 6:**
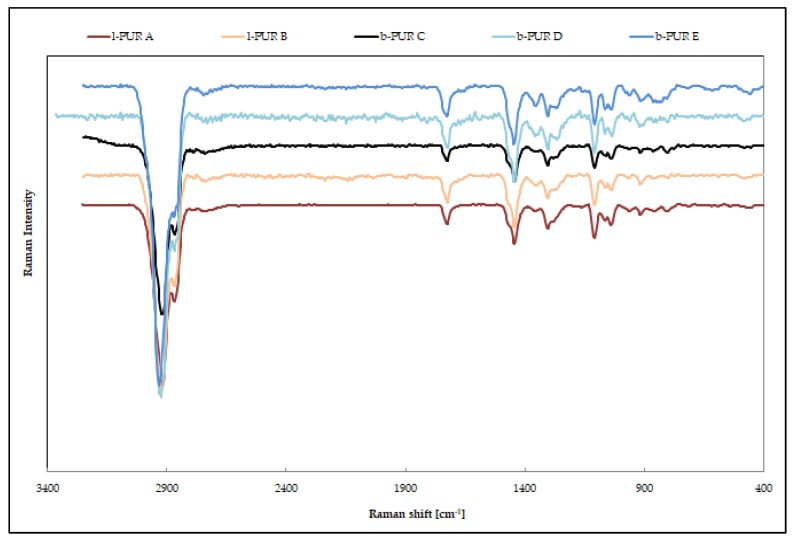
Raman spectra of linear and branched PURs.

The very strong FTIR spectra band at about 1740–1720 cm^−1^, ascribed the free C=O ester groups, was quite weak in Raman spectra of both types of PURs (Figure 6). In addition. because of polarity of urethane, the N–H presence on Raman spectra was invisible. The largest bands were asymmetric at 2930–2917 cm^−1^ and symmetric at 2890–2863 cm^−1^, with CH_2_ stretching vibration and CH_2_ bending vibration at ≈1448 cm^−1^.

**Figure 7:**
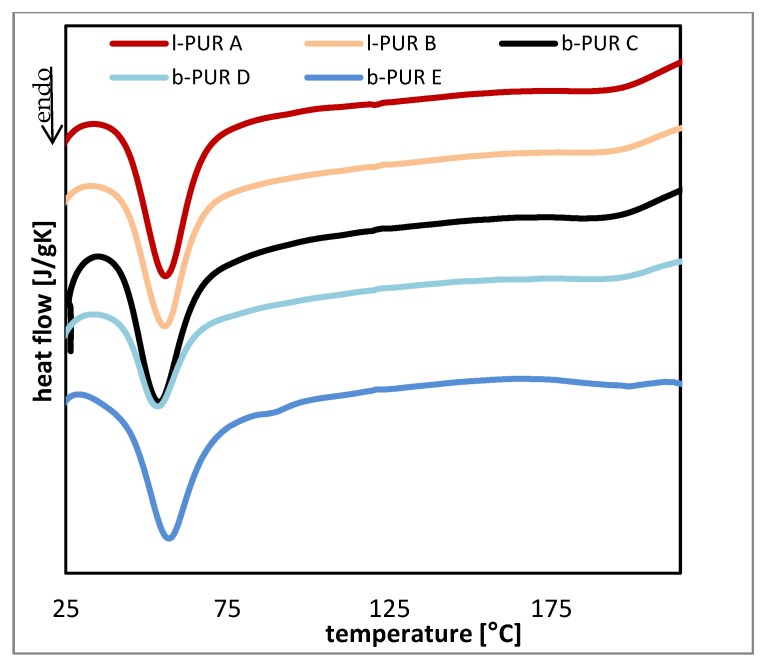
Differential scanning calorimetry (DSC) thermograms of linear and branched PURs.

DSC thermograms of investigated samples were typical for segmented PURs. Two endotherms, ascribed to the melting crystalline phase of soft (with melting temperature *T*_m1_) and hard (with melting temperature *T*_m2_) segments were visible on DSC diagrams of linear and branched PURs (Figure 7). The data read from thermograms are shown in Table 2.

Using semicrystalline PCL to build soft segments caused obtained PURs to be characterized by partial crystallinity of soft segment domains.

Total melting enthalpies (Δ*H*) of both soft and hard segments of linear PURs were higher than branched PURs, which suggested that linear PURs were more crystalline than branched ones, as it was expected.

Melting temperatures of soft and hard segments depended on segments separation. In case of linear PURs (l-PUR A and l-PUR B) and branched b-PUR E (with low amount of PCL_triol_) T_m1_ was closer to melting temperature of PCL_diol_ (*T*_m_ = 60.1 °C) than for b-PUR C and b-PUR D, suggesting the good separation of soft segment chains. However, the endothermic peak ascribing melting of crystalline phase of soft segments on DSC thermograms of b-PUR E was very wide and was integrated to baseline, including a small peak about 88 °C. It was concluded that this broad peak represents melting of all kinds of spherulites with different volume that formed in range of soft segments. This was a reason of high Δ*H*_1_ b-PUR E.

Mobility of long aliphatic chains of PUR in its structure facilitated re-organization of PCL and, in consequence, formation of crystals. The presence of amorphous R,S-PHB did not restrict a tendency of PCL to crystallize. A different situation was observed for branched PURs. In the case of reduction of chain mobility via branch nodes, partial replacement of PCL_diol_ chains by amorphous R,S-PHB reduced crystallinity of soft segments. In b-PUR E with a very small amount of trifunctional PCL_triol_ the tendency was the same as in linear samples. This dependence of crystallinity with the number of branch was also observed by Mahapatra et al. in hyperbranched PURs [15].

What was interesting was the presence of R,S-PHB in the PURs structure reduced melting enthalpy of hard segments. It was supposed that the presence of the lateral methyl group in R,S-PHB hindered ordering of the PUR chain. As mentioned before the presence of non-bonded C=O in PURs containing R,S-PHB was confirmed by FTIR and contribution of C=O urethane groups in formation of hydrogen bonds could not be determined. However, reduced crystallinity of hard segments (lowered Δ*H*_2_) suggested the absence of hydrogen bonds between urethane groups and connected with it chains ordering.

**Figure 8:**
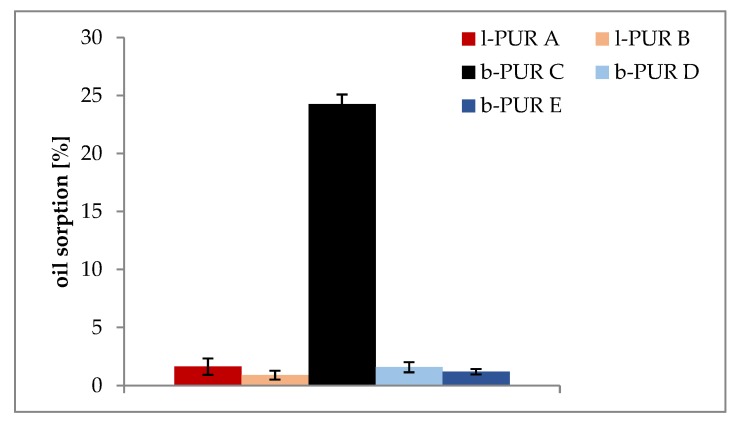
Oil sorption by linear and branched PURs.

Architecture of PURs structure and their hydrophilicity also influenced tendency to oil sorption. The data presented in Figure 8 indicated that branched PUR without R,S-PHB absorbed more vegetable oil than linear l-PUR A. The presence of free spaces in the polymer network of branched PURs, which were a result of the presence of branching points, ought to facilitate migration of oil molecules. However, the ability of investigated PURs to sorb oil was also connected with their soft segment building. Soft segments, both linear and branched PURs, were mainly built with chains of hydrophobic polycaprolactone. Oil sorptions of l-PUR A and b-PUR C were higher than appropriate PURs containing R,S-PHB in their structure. Increasing PURs hydrophilicity after using R,S-PHB to build soft segments was observed earlier [7]. In consequence, branched b-PUR C with soft segments built only with polycaprolactone chains and characterized by the lowest density absorbed highest amount of lipid among all investigated PURs.

**Figure 9:**
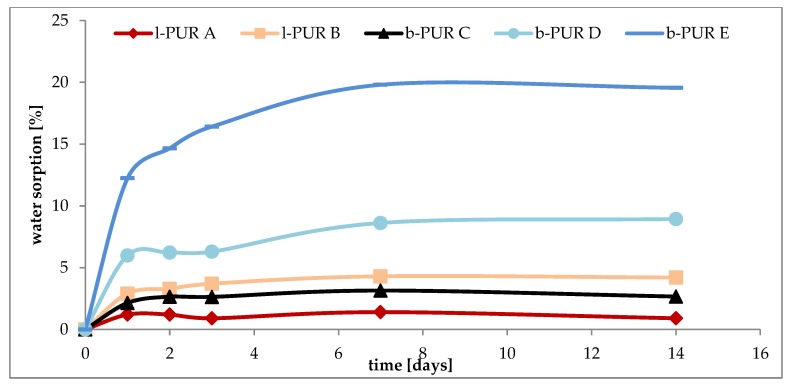
Water sorption by linear and branched PURs.

The water sorption is a synergistic effect of crystallinity and the chemical structure of chains in PURs. Introducing R,S-PHB into the PURs (both linear and branched) structure, instead of hydrophobic polycaprolactone chains, increased their hydrophilicity (Figure 9), as it was observed earlier [16]. Crystallinity of samples and the presence of free spaces in polymer network were very important for water sorption. Comparing the structure of l-PUR A and b-PUR C, it could be said that their chemical building was very similar to each other. They differed only in architecture, i.e., the presence of long, side chains in the branched PUR structure resulting from the introduction of tri-functional PCL_triol_ (Figure 1). In consequence, the free spaces in the b-PUR C structure were formed, which facilitated migration of water molecules into polymer bulk. However, as it was said before, more important was using amorphous, hydrophilic R,S-PHB as a part of soft segments. Its presence significantly increased the ability of investigated PURs to absorb water. Branched b-PUR E with the high amount of R,S-PHB and with very low crystallinity of hard segments absorbed the most water (Figure 9).

## 4. Conclusions

Aliphatic PURs with synthetic poly([R,S]-3-hydroxybutyrate) (R,S-PHB) in soft segments were synthesized with the prepolymer method. Branched and linear PURs differed in architecture of structure even where they were based on the same chemical structure of starting substrates.

The spectroscopies analysis (FTIR and Raman spectra), used for structure estimation, indicated that urethane linkage appeared and isocyanate peaks related 4,4’-methylene dicyclohexyl diisocyanate (H_12_MDI) disappeared in all the samples after the synthesis. All N–H groups were engaged in formation of hydrogen bonds in linear PURs, whereas in branched PURs the presence of non-associated N–H groups was confirmed. Introducing R,S-PHB into the structure of branched PURs inhibited a tendency of urethane groups to hydrogen bonding. Introducing a small amount of R,S-PHB did not change crystallinity of linear PURs, but reduced melting enthalpy of branched PUR. Significant changes of sorptive properties (increased water sorption and decreased oil sorption) were observed for both linear and branched PURs after incorporation of R,S-PHB as a soft segments building block into the macromolecular structure. Polymer materials with the desired sorption characteristic can be obtained by manipulating architecture of the PUR structure and the amount of added synthetic R,S-PHB.

## Figures and Tables

**Table 1:** The code of linear and branched polyurethanes (PURs) and amounts of reactants [g] in their synthesis (calculated for 100 g of final polymer).

PUR		PCL_triol_	PCL_diol_	R,S-PHB	H_12_MDI	1,4-BD
linear	l-PUR A	-	74.62	-	21.66	3.72
	l-PUR B	-	59.75	14.96	21.56	3.72
branched	b-PUR C	5.50	66.13	-	24.10	4.27
	b-PUR D	5.51	44.69	21.85	23.90	4.05
	b-PUR E	2.20	35.24	35.20	23.28	4.08

**Table 2:** Melting temperature, enthalpy of soft [*T*_m1_, Δ*H*_1_] and hard [*T*_m2_, Δ*H*_2_] segments and density (with standard deviation SD) of PURs.

PUR	Density ± SD [g/cm^3^]	*T*_m1_ [°C]	Δ*H*_1_ [J/g]	*T*_m2_ [°C]	Δ*H*_2_ [J/g]
l-PUR A	1.16 ± 0.06	55.6	53.2	195.7	17.1
l-PUR B	1.13 ± 0.02	55.3	54.4	194.1	10.4
b-PUR C	1.02 ± 0.08	53.6	46.2	194.3	11.4
b-PUR D	1.15 ± 0.02	53.2	30.4	196.2	5.5
b-PUR E	1.17 ± 0.03	56.6	46.7	198.8	1.7

## Supplementary Materials

The supplementary materials are available online at http://www.mdpi.com/2073-4360/10/8/826/s1.
