# Supplementary figures and images for: Branched Polyurethanes Based on Synthetic Polyhydroxybutyrate with Tunable Structure and Properties
Source: Polymers (Basel). 2018 Jul 26;10(8):826. doi: 10.3390/polym10080826 (PMC6404007; doi:10.3390/polym10080826)

**Figure S1.** FTIR spectrum of R,S-PHB

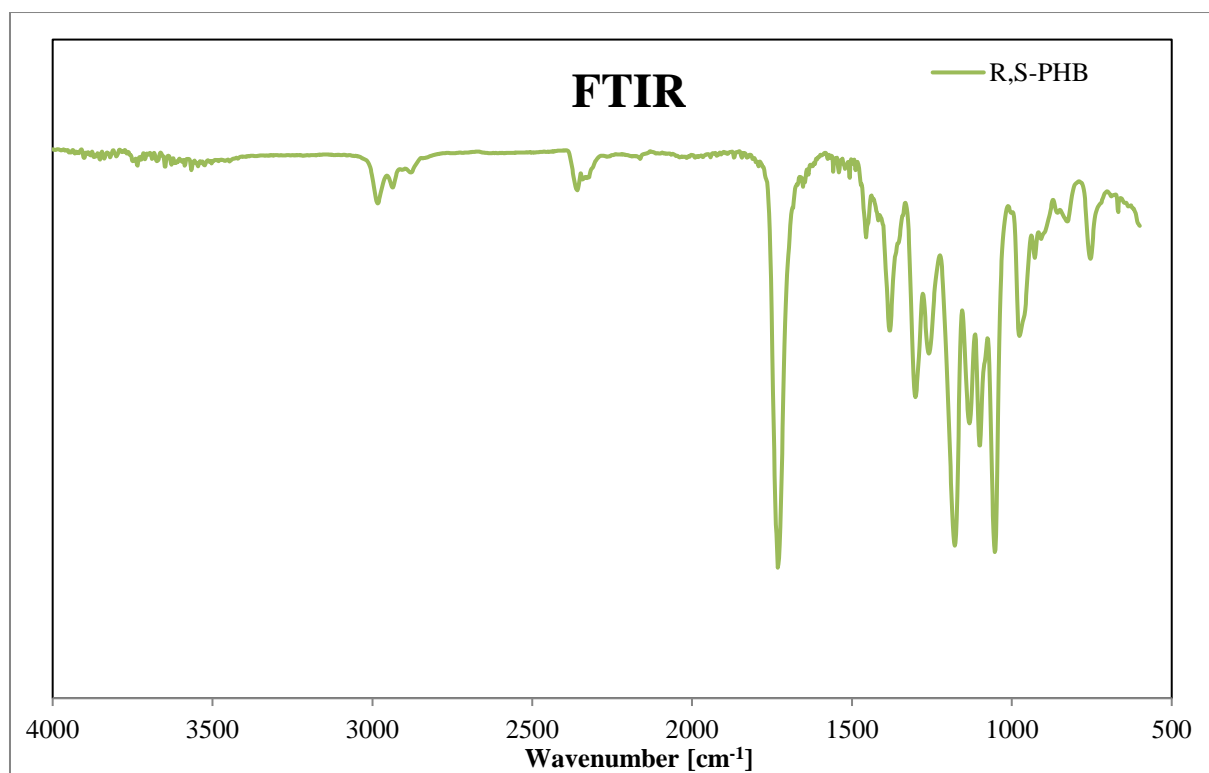

Supplement: Supplementary file 1 [file polymers-10-00826-s001.pdf]
